# Primary lymphoma of the breast involving both axillae with bilateral breast carcinoma

**DOI:** 10.1186/1477-7819-6-52

**Published:** 2008-05-20

**Authors:** Neeraj K Garg, Nitin B Bagul, Gary Rubin, Elizabeth F Shah

**Affiliations:** 1 Department of Surgery, Conquest Hospital, UK; 2 Department of Surgery, University Hospital of North Tees, UK; 3 Department of Radiology, Royal Sussex County Hospital, UK

## Abstract

**Background:**

Primary Non-Hodgkin's Lymphoma (PHNL) of the breast is a rare entity, while secondary involvement of the breast with diffuse disease of Non-Hodgkin's lymphoma (NHL) is more common. However, PNHL is the most frequent haematopoietic tumour of the breast. Diagnostic criteria for PNHL of the breast are presence of technically adequate pathologic specimens, close association of mammary tissue and lymphomatous infiltrate, no prior diagnosis of an extarammamary lymphoma, and no evidence of concurrent widespread disease, except for ipsilateral axillary lymph nodes if concomitant with the primary lesion.

**Case presentation:**

A 57-year-old woman was recalled because her screening mammograms revealed three separate lesions in her right breast and one in the left. Histology of the lesions confirmed lymphoma in one breast with ductal carcinoma in the other.

**Conclusion:**

Most of reported cases in literature have been involving the right breast, and almost all the patients were females. NHLs of the breast typically present as unilateral mass; the frequency of bilateral disease at first presentation ranges from 5–25%. Our objective is to report a case of primary lymphoma of the breast involving both axillae with concomitant bilateral primary breast cancer which has not been reported yet to our best of knowledge in literature.

## Background

About 50% of lymphomas are primary extranodal non-Hodgkin's lymphomas (NHL) [1]. Primary non-Hodgkin's lymphoma (PNHL) of the breast is a rare entity, while secondary involvement of the breast with diffuse disease of NHL is more common [2]. However, PNHL is the most frequent haematopoietic tumour of the breast [3]. Diagnostic criteria for PNHL of the breast are [4]: 1) presence of technically adequate pathologic specimens, 2) close association of mammary tissue and lymphomatous infiltrate, 3) no prior diagnosis of an extarammamary lymphoma, and 4) no evidence of concurrent widespread disease, except for ipsilateral axillary lymph nodes if concomitant with the primary lesion.

The number of cases of PNHL of the breast reported to date is around 250 [5]. Most of these cases have involved the right breast, and almost all the patients were females. NHLs of the breast typically present as unilateral mass; the frequency of bilateral disease at first presentation ranges from 5–25% [6]. To the best of our knowledge a case of primary lymphoma of the breast involving both axillae with concomitant bilateral primary breast cancer has not been reported yet.

## Case presentation

A 57-year-old woman attended for routine mammography screening. She was recalled because her screening mammograms revealed three separate lesions in her right breast and one in the left. At clinical examination, there were only two vaguely palpable masses in the upper outer quadrant of her right breast and one in the upper outer quadrant of her left breast. Overlying skin was normal and no regional lymph nodes were palpable.

Mammography of the left breast revealed a 15-mm lesion in the upper outer quadrant and on core biopsy it was shown to be a grade I invasive ductal carcinoma which was oestrogen and progesterone receptor positive. On the right side, the first mass, an area of about 13-mm of micro calcification, was situated in the upper outer quadrant at the 10'o clock position, and on core biopsy was confirmed as a grade I invasive ductal carcinoma, also both oestrogen and progesterone receptor positive. The second mass was at the 11'o clock position and the third was in the lower inner quadrant and these latter two lesions on core biopsy showed lymphoma-like features but not breast carcinoma. The core biopsies were therefore sent for expert opinion but this supplementary report was also not conclusive. Therefore, diagnostic excision biopsy was recommended. In the meantime, staging computerised tomography of her chest and abdomen was performed and was found to be normal. Treatment options were then openly discussed with the patient and her family and she opted for a right mastectomy and axillary node clearance and wide local excision and axillary node sampling on the left. She felt this would be easier than a combination of therapeutic local excision of the cancers and diagnostic needle localisation of the other masses in the right breast.

**Figure 1:**
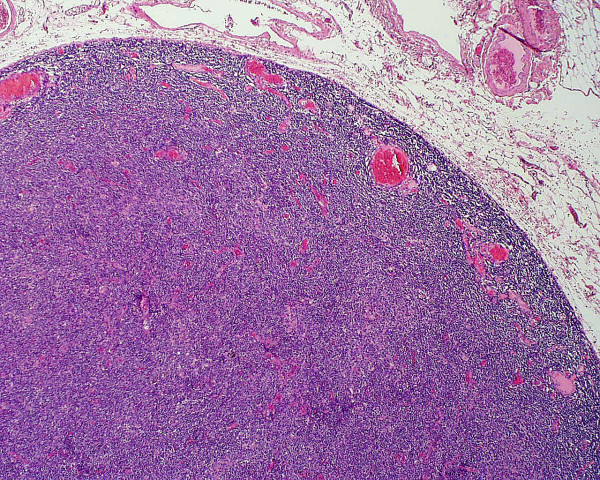
Photomicrograph of histology specimen of an invasive ductal carcinoma shows that it extends irregularly through the tissue as cords and nests of neoplastic cells with intervening collagen. It has pleomorphic cells infiltrating through the stroma. Note the abundant pink collagen bands from desmoplasia, making the tumor feel firmer than normal breast tissue on palpation.

**Figure 2:**
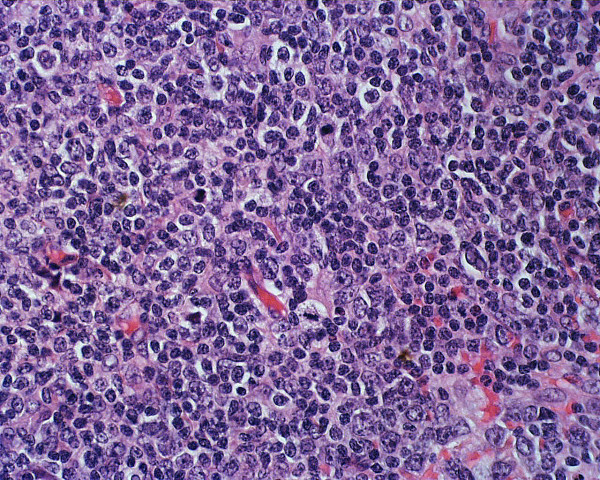
The low power view of the node shows how the normal follicular architecture has been effaced.

**Figure 3:**
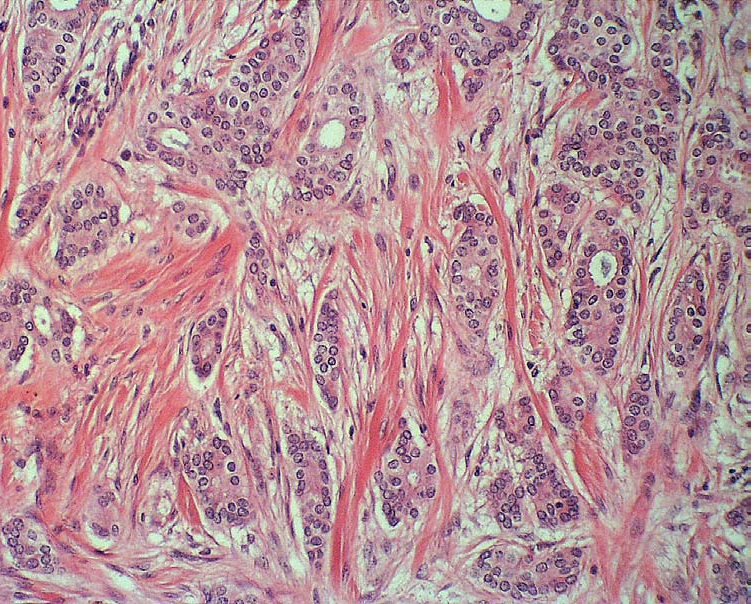
The high power view shows the abnormal lymphoid population of small to medium sized centrocyte-like B-cells and monocytoid B-cells.

Definitive histopathology supplemented by immunohistochemistry was compatible with marginal zone B-cell lymphoma of right breast and involving the lymph nodes of both axillae (Figure 1, 2). In addition, it also confirmed in the right breast an 8 mm, grade I invasive ductal carcinoma (Figure 3) with intermediate grade ductal carcinoma in situ and a 12 mm grade I invasive ductal carcinoma on the left side. There was no lymphatic spread of breast cancer into either axilla. Her breast cancers were treated by radiotherapy to her conserved left breast and adjuvant hormonal therapy. The patient was referred by the breast team to the specialist lymphoma team but they did not recommend any further treatment for the Nodal marginal zone B-cell lymphoma as it was indolent tumour and they deemed it had been adequately treated by the surgery alone. She has follow up in six monthly intervals.

## Discussion

Non-Hodgkin's lymphoma may originate in, or spread to, any extranodal organ. Breast lymphoma is a rare disease, either as a primary site or as secondary involvement, representing 0.04–0.5% of malignant breast tumours [7]. It is almost always of non-Hodgkin's type. Secondary involvement of the breast in patients with diffuse disease is more common [8].

Most patients with primary lymphoma of the breast develop distant disease to other regions. Within the breast, the most common primary lymphomas are B cell (more rarely T cell) non-Hodgkin's lymphoma. They appear at an elderly age with focal or diffuse localization and usually they are unilateral. Early diagnosis is crucial for clinical outcome [9].

Lymphomas are a distinct possibility in the diagnosis of breast tumours. PNHL of the breast remains a diagnosis of exclusion, and the diagnosis cannot be made without a very thorough evaluation [4]. If a patient presents with a rapidly growing breast tumour, lymphoma should be considered before any surgical intervention is performed. Early decision is vital considering the aggressive nature of the lesion and the prognosis. A high index of suspicion and an understanding of the clinical behaviour of PBL are necessary for proper patient management [7].

The most common symptoms of breast lymphoma are a painless breast mass, most frequently located in the outer quadrants [10]. Skin retraction, erythema, peau d'orange appearance, and nipple discharge are uncommon in lymphomas [11]. In 50% of cases ipsilateral axillary node involvement is present.

A distinct mammographic or sonographic pattern has not been reported in the literature because primary lymphoma shows no specific characteristics which differentiate it from other benign and malignant breast disorders [8]. Mammography usually demonstrates a well-circumscribed, uncalcified mass with sharp or minimally irregular margins [12].

On Magnetic resonance imaging (MRI) primary lymphoma is more commonly visualized as a lobulated lesion with expansive and infiltrating features. MRI findings are non-specific: in the literature, patterns of primary lymphoma with variable signal intensity and morphology have been reported [2]. Despite its non specific signs, MRI plays a major role in the determination of the extent and number of lesions, and in the evaluation of cutaneous, subcutaneous and nodal involvement of the contralateral breast.

The definitive diagnosis is therefore histological and allows the planning of surgery (lesion removal) or medical therapy (chemotherapy +/- radiotherapy). Both clinical stage and histological subtype of the lymphoma appear to be important in determining the prognosis of breast lymphomas.

## Conclusion

We report a highly unusual case of primary lymphoma of the breast involving both axillae associated with bilateral invasive ductal carcinoma that was successfully treated with surgery, radiotherapy to the breast and hormonal therapy.

## Competing interests

The authors declare that they have no competing interests.

## Authors' contributions

NKG and NBB drafted the manuscripts. GR and EFS critically reviewed and improved the manuscript. All authors read and approved the final manuscript.

## Consent

Written informed consent was taken from the patient for publication of this report
